# Fault Detection on the Edge and Adaptive Communication for State of Alert in Industrial Internet of Things

**DOI:** 10.3390/s23073544

**Published:** 2023-03-28

**Authors:** Yuri Santo, Roger Immich, Bruno L. Dalmazo, André Riker

**Affiliations:** 1 Institute of Exact and Natural Sciences (ICEN), Federal University of Pará, Belém 66075-110, Brazil; 2 Metropole Digital Institute (IMD), Federal University of Rio Grande do Norte (UFRN), Natal 59078-970, Brazil; 3 Computer Science Center (C3), Federal University of Rio Grande, Rio Grande 96203-900, Brazil

**Keywords:** Industrial Internet of Things (IIoT), machine learning, edge computing

## Abstract

Industrial production and manufacturing systems require automation, reliability, as well as low-latency intelligent control. Industrial Internet of Things (IIoT) is an emerging paradigm that enables precise, low latency, intelligent computing, supported by cutting-edge technology such as edge computing and machine learning. IIoT provides some of the essential building blocks to drive manufacturing systems to the next level of productivity, efficiency, and safety. Hardware failures and faults in IIoT are critical challenges to be faced. These anomalies can cause accidents and financial loss, affect productivity, and mobilize staff by producing false alarms. In this context, this article proposes a framework called Detection and Alert State for Industrial Internet of Things Faults (DASIF). The DASIF framework applies edge computing to execute highly precise and low latency machine learning models to detect industrial IoT faults and autonomously enforce an adaptive communication policy, triggering a state of alert in case of fault detection. The state of alert is a pre-stage countermeasure where the network increases communication reliability by using data replication combined with multiple-path communication. When the system is under alert, it can process a fine-grained inspection of the data for efficient decison-making. DASIF performance was obtained considering a simulation of the IIoT network and a real petrochemical dataset.

## 1. Introduction

Machine learning (ML) has been largely agreed upon as a key building block for the Industrial Internet of Things (IIoT). ML can support intelligent and quick decisions needed in IIoT environments [1]. Without fast and precise detection and decision-making, manufacturing is susceptible to all sorts of delays, which can cause financial loss and may represent a security threat.

In IIoT systems, the benefits of ML are potentiated by the capabilities of edge computing. By definition, edge computing has emerged as a paradigm to increase the computational power of IoT systems, providing extra storage and processing [2]. Edge computing devices are deployed near IoT devices to reduce the time necessary to complete computationally demanding tasks.

Hardware failures and faults in IIoT are critical challenges to be faced. A vast set of faults can occur in IIoT devices, including drift, spike, stuck, offset, bias, gain, out-of-bounds, saturation, and precision degradation [3]. All these anomalies can cause accidents, halt production, and mobilize staff by producing false alarms.

To mitigate the problems caused by IIoT faults, the next generation of IIoT systems requires: (i) precise and early fault detection; and (ii) autonomic response and reaction. The first requirement demands constant monitoring of the produced data, and the second, not least important, consists of autonomously reacting when the fault has been detected. In this context, modern industrial business models consider that solving a fault in its early stages prevents collateral damage and results in fewer costs.

Many works in the literature propose solutions to meet the requirements of IIoT. Most of them are based on ML classifiers to support precise fault detection. Only some works seek to minimize the response time during the detection. Additionally, a reduced number of works provides any autonomic reaction for detected faults. To fill this gap, this article proposes a framework called Detection and Alert State for Industrial Internet-of-Things Faults (DASIF). The DASIF framework applies highly precise and low latency machine learning models to detect IIoT faults and also autonomously enforce a state of alert in case of detecting the fault. The faults are double classified on the edge computing devices using a decision tree and Gaussian naive Bayes. The state of alert enforced by DASIF is the first-stage countermeasure for fine-grained data analysis. Under this state, the network decreases the communication interval and uses data replication and multiple-path communication to achieve higher communication reliability. In our previous work [4], we conducted a study to select the best ML models for the DASIF framework. In [4], we evaluated six machine learning classifiers measuring accuracy, precision, recall, F1 score, training time, and response time.

The contributions of this article are the following:

- to propose an ML-based framework running on edge computing for detecting Internet of Things (IoT) faults in industrial environments;
- to present a Markov chain-based algorithm to inject a set of faults into IIoT datasets;
- to enforce an adaptive communication policy, instituting a state of alert as an autonomic reaction when a fault has been detected.

The rest of this paper is organized as follows. Section 2 presents the related work. Section 3 presents the proposed framework, and Section 4 introduces the evaluation scenario, settings, and the obtained results. In the end, Section 5 presents the conclusions and potential directions for future research.

## 2. Related Work

A vast literature applies machine learning models to support smart decisions in industrial IoT environments. Most of these works propose solutions for anomaly detection related to security aspects. A reduced number of works aims to detect anomalies in the sensed data produced by IoT devices and caused by internal hardware and software malfunctions or external factors, such as vibration.

To detect faults and errors in IoT devices using machine learning classifiers, Jan et al. [5,6] propose a diagnosis system to detect sensor fault. The authors consider devices with limited computation resources, such as memory, processing, and energy. This solution is distributed and based on a Support Vector Machine (SVM) model, where response time is not the top priority for the application.

Saeed et al. [7] analyze different classifiers using a dataset with drift fault injection. The faults were injected using real data from a digital relative temperature/humidity sensor (DHT22) and an Arduino controller. For detection, a Raspberry Pi is used. The performance was measured using a large set of machine learning models: SVM, ANN, naive Bayes, KNN, and decision tree. The authors compare the performance of the machine learning models in terms of precision, recall, F1 score, and total accuracy.

Javaid et al. [3] aim to detect and diagnose faults based on decision fusion with different classification techniques named: Enhanced K-Nearest Neighbor (EKNN), Enhanced Extreme Learning Machine (EELM), Enhanced Support Vector Machine (ESVM), and Enhanced Recurrent Extreme Learning Machine (ERELM). The authors consider the offset, gain, stuck, and out-of-bounds faults.

Zidi et al. [8] apply machine learning for fault detection in wireless sensor networks. The solution considers an SVM model for dealing with offset, gain, stuck-at, and out-of-bounds faults in a real dataset from the University of North Carolina at Greensboro [9].

In a nuclear power plant scenario, Naimi et al. [10] propose fault detection and diagnosis based on neural networks and a KNN algorithm applied to a pressurized water reactor. First, the neural network performs detection. Second, the KNN algorithm classifies the faults. The KNN performance is also compared to neural networks and SVM. This work includes bias, drift, actuator offset, and saturation faults.

Khodabaksh et al. [11] consider a method for real-time data validation, gross error detection, and classification. This work is based on data from petrochemical power plants of an oil refinery. The injection of bias, drift, and precision degradation failures was based on statistical studies of sensor data and the observation of changes in mean and variances. The classification relies on the complex decision tree, neural network, and KNN algorithms. The performance was measured using precision and recall. The authors made available the dataset for academic use [12].

So far, all the mentioned works do not consider any countermeasure to be applied after the faults have been detected. Dofe et al. [13] present a comprehensive perspective on countermeasures against IoT attacks. Mustafa et al. [14] propose three countermeasures to preserve privacy when abnormal behavior is detected in assisted living applications.

Summing up, many works explore fault detection through the SVM classifier as can be observed in Jan et al. [5,6] and Zidi et al. [8]. However, an industrial scenario requires a sensitive time application to deal with faults, but the SVM demands higher computation time. Naimi et al. [10] also do not consider the necessary time to apply a two-phase solution. Additionally, how to react after the fault has been detected is an important aspect, but countermeasures are often considered only from the security perspective. Therefore, there is a lack of novel solutions capable of detecting and reacting against possible faulty situations in IIoT environments.

## 3. Detection and Alert State for Industrial Internet of Things Faults (DASIF)

The main goal of the proposed framework is to provide fast and precise fault detection and reaction in industrial internet of things devices. The rest of this section is organized as follows. Section 3.1 details the industrial scenario and the overview of the proposed solution. Section 3.2 introduces the machine learning classifiers used in DASIF. Section 3.3 presents the details of the industrial dataset and how the faults have been injected. Section 3.4 describes the adaptive communication policy that sets a state of alert when a fault has been detected.

### 3.1. Overview and Proposed Framework

**Figure 1:**
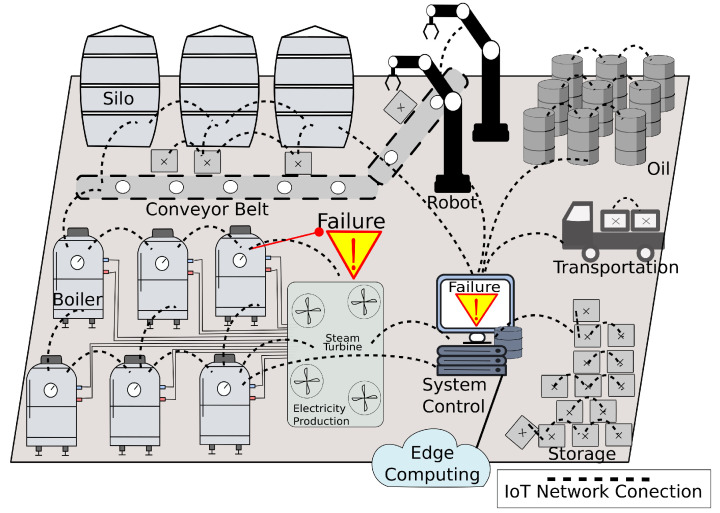
Industry scenario.

Figure 1 illustrates an industrial environment with silos, conveyor belts, boilers, pipes, turbines, energy production, storage boxes, vehicles, robots, and oil barrels. All these objects and machinery are monitored and actuated by a set of IoT devices, which send and receive traffic via a wireless network to the system control.

Typically, the IoT network traffic feeds the system control with the pressure in the pipes, the turbines, rotation, the vehicles, position, the temperature of the boilers, the speed of the conveyor belt, and many other data.

The system control offloads the IoT data to edge computing since it provides cloud services near the network devices, supporting high computational power and low delay. The processing power in the edge is capable of analyzing a massive amount of IoT traffic and providing multiple data services, including intrusion and fault detection. Edge computing can successfully apply algorithms to support dynamic resource allocation, e.g., processing and memory, to meet energy or response time requirements.

**Figure 2:**
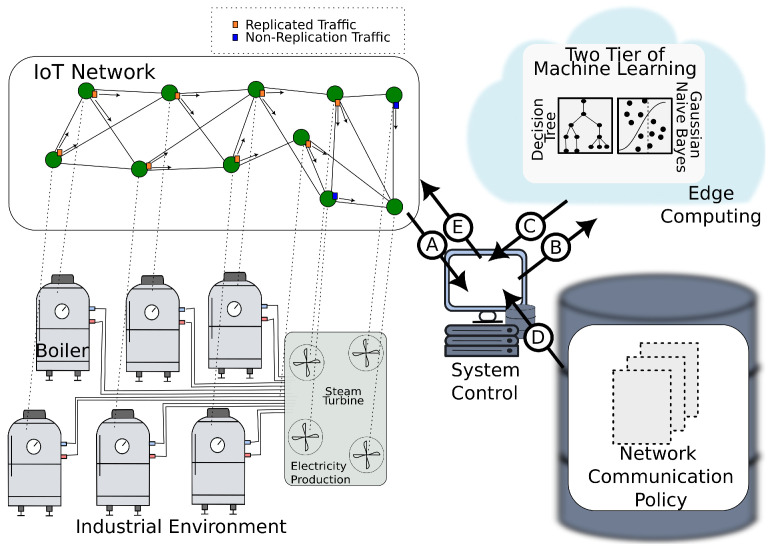
Detection and Alert State for Industrial Internet of Things Faults (DASIF) framework.

In this context, the proposed framework, called Detection and Alert State for Industrial Internet of Things Faults (DASIF), is a solution designed to detect IoT faults in industrial environments. As shown in Figure 2, the DASIF framework has a system control in which IoT traffic is inspected and forwarded to the edge. Additionally, the system control provides data visualization and issues commands for the automation of the factory.

The IoT devices deployed in an extensive number support monitoring, automation, and communication. However, these devices are prone to errors and faults, which distort the measured values. For instance, IoT devices can abruptly stop monitoring the target, sending invalid pressure values in a pipe or a valve. These errors and faults can cause severe damage to manufacturing, including accidents, financial loss, and false alarms.

In this framework, edge computing provides fast processing of machine learning classifiers. Based on the study performed in our previous work [4], DASIF relies on a two-tier machine learning for precise and fast IoT fault detection. The fault classification is double-checked using a decision tree and Gaussian naive Bayes.

Another component of this framework is the repository of communication policy. In this repository, the network communication settings are defined for a set of situations the industrial system must deal with. In this work, DASIF foresees normal and alert policies. A detected IoT fault triggers the alert policy. Under an alert policy, the IoT network communicates using data replication and with more intense traffic production. This reaction enables the system to perform fine-grained checks on the error and provide information for critical decision-making, including triggering an unmanned aerial vehicle (UAV) to visit the location where the alert is being issued to verify in loco the hardware conditions and make adjustments to the IoT devices.

To achieve IoT fault detection, DASIF executes five tasks as indicated by the labels in Figure 2 and are described as follows.

- **Label A:** The system controller receives and inspects the traffic produced by the deployed IoT network.
- **Label B:** The relevant data for fault detection are sent to the edge.
- **Label C:** The machine learning output containing the fault classification is sent back to the control system.
- **Label D:** The system control selects an appropriate communication policy according to the classification received from the machine learning models.
- **Label E:** The control system enforces the communication policy, issuing messages to the nodes.

### 3.2. Machine Learning Classifiers for Fault Detection

DASIF has a machine learning layer that detects the faults of IoT devices. This layer applies a double classification using a decision tree and Gaussian naive Bayes (GNB). Decision trees are a top-down, divide-and-conquer approach to supervised classification. At the beginning of the training phase, all training samples are assigned to the root node. These training samples are partitioned and assigned to child nodes to increase the purity of the resulting child nodes. The procedure is repeated at each node until the leaf nodes have training examples of a single class, i.e., the leaf nodes are pure [15]. A naive Bayesian classifier is a simple and efficient classifier based on the Bayesian theory. However, it is well known that NBC is based on the assumption that all attributes are independent of each other [16]. A Gaussian naive Bayes classification is a case of the naive Bayes method with an assumption of having a Gaussian distribution of attribute values given the class label.

The single classifier has a limited ability to deal with the problem of a larger amount of data. Multiple classifier systems can improve the performance of a single classifier, especially in critical systems such as industrial internet of things. In DASIF, the combination of these two machine learning models assures that decision-making is based on two distinct classifiers, increasing diversity in assumptions and training processes. The DASIF final decision is Fault or Normal. DASIF output is a fault if any of the classifiers produces a fault classification.

### 3.3. Dataset Description and Fault Injection

This article uses the IIoT dataset from Khodabakhsh et al. [11], which provides real-world measurements from over 1000 devices deployed at Turkish Petroleum Refineries Inc. (TUPRAS) power plants. The TUPRAS dataset is a sample of real-world data measured every minute and is available for academic use at [12].

This petrochemical dataset contains 200,000 flow sensor records (water, superheater, steam) sampled every 60 s in the TUPRAS power plant for approximately 5 months. These data are replicated 5 times to form 1 million rows to represent actual sensor loads better. Each row has records from 3 flow sensors in the power plant dataset and 17 flow sensors in the petrochemical dataset.

According to [5], faults are defined as deviations from expected behavior in the device output. The faults are data corruption behavior, which is related to the physical defects of the devices and their operational conditions.

**Figure 3:**
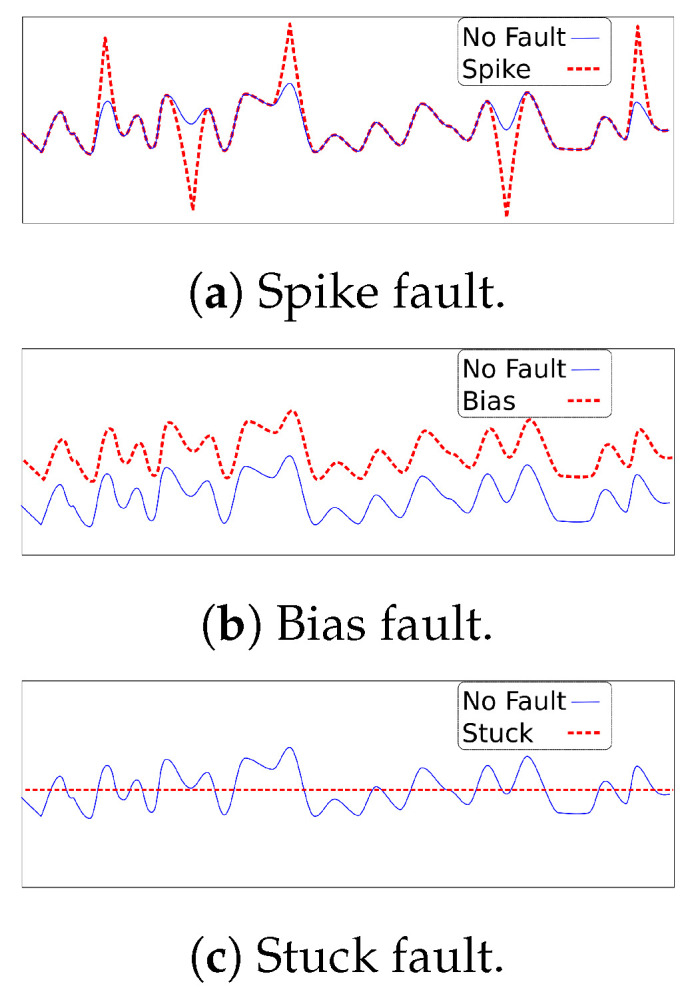
Illustration of faults.

In this paper, three faults have been considered, namely spike, stuck, and bias. As shown in Figure 3a, a spike fault is an effect observed as a large-amplitude value occurring at time intervals in the sensor output. Figure 3b illustrates the bias fault. In this fault, a shift from the normal value is observed since a constant value is added to the normal output. A stuck fault (see Figure 3c), also called a complete fault, blocks the sensor output at a fixed value. This fault can be temporary or permanent.

The original TUPRAS dataset does not identify, i.e., label, any faults or errors. However, machine learning models that use supervised methods require labeled data to identify errors. Based on a statistical dataset analysis, we have developed Algorithm 1 to inject faults into the dataset and the appropriate labels for training and testing.

This algorithm is responsible for injecting stuck, bias, and spike faults. In order to achieve this goal, the ratio between the maximum value (l. 5) and the mean (l. 4) of the dataset is used. This ratio (l. 6) represents the percentage of max increase to the mean. Aiming to replicate the fault behavior, the functions presented in the algorithm deal with different calculations based on the same ratio, replacing some dataset values with fault values according to the Markov chain. The stuck function performs a decrease in the sensed value (l. 14) and becomes constant in the subsequent minutes. While the fault lasts, the bias function executes an increase in the sensed value (l. 29). The spike function performs increment (l. 44) and decrement (l. 47). These functions return a modified dataset with faults.

**Figure 4:**
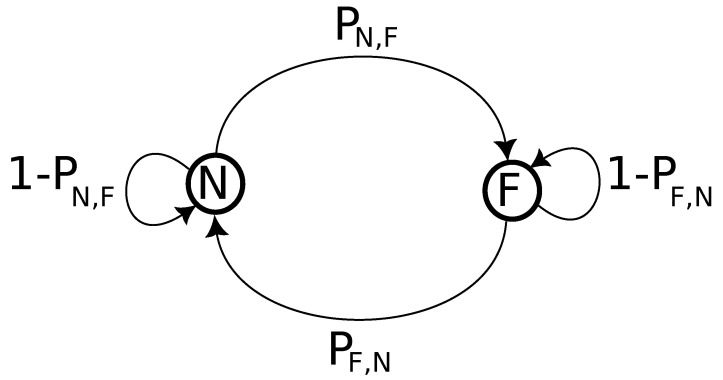
A two-state Markov chain to inject faults.

As can be observed, Algorithm 1 relies on Markov chain to determine the distribution of faults along the dataset time series. A two-state Markov chain has been used for this algorithm, as illustrated in Figure 4. The N and F denote the normal and fault behavior states, respectively. The states can be maintained, or the transitions between states can occur according to a defined probability.
**Algorithm 1:** Fault Injection Algorithm1:**Input**: original dataset.2:**Output**: dataset with fault injection.3:**Start**4:    mean ← dataset mean5:    max ← dataset maximum value6:    ratio ← max / mean7:    8:    **function** stuck (dataset, ratio)9:          stuckRate ← ratio10:        stuckTemp ← 011:        **for** i=1:dataset size **do**12:           **if** index i is in Markov chain **then**13:               **if** stuckTemp == 0 **then**14:                   stuckTemp ← ith value * (2-stuckRate)15:                   ith value of dataset ← stuckTemp16:               **else**17:                   ith value of dataset ← stuckTemp18:               **end if**19:           **end if**20:        **end for**21:        **return** dataset22:    **end function**23:    24:    **function** bias (dataset, ratio)25:        biasRate ← ratio26:        biasTemp ← 027:        **for** i = 1:dataset size **do**28:           **if** index i is in Markov chain **then**29:               biasTemp ← ith value * biasRate30:               ith value of dataset ← biasTemp31:           **end if**32:        **end for**33:        **return** dataset34:    **end function**35:    36:    **function** spike(dataset, ratio)37:        spikeRate ← ratio38:        spikeTemp ← 039:        random ← 040:        **for** i = 1:dataset size **do**41:           **if** index i is in Markov chain **then**42:               random ← random integer number between 0 and 143:               **if** random == 0 **then**44:                   spikeTemp ← ith value * spikeRate45:                   ith value of dataset ← spikeTemp46:               **else**47:                   spikeTemp ← ith value * (2-spikeRate)48:                   ith value of dataset ← spikeTemp49:               **end if**50:           **end if**51:        **end for**52:        **return** dataset53:    **end function**54:**End**

### 3.4. Network Reaction Policy for Detected Faults

DASIF is designed to be autonomic since an IoT network deployed in an industrial environment requires the management of hundreds or thousands of devices. Being autonomic means to be self-sufficient or self-healing, and self-protective. The main idea is to provide fast resource management of the system with low or no human intervention.

To achieve that, DASIF has two network communication policies, called normal and alert policies. The normal is applied when no fault has been detected. The alert policy is enforced after any fault has been detected, triggering a state of alert in the system, which is an early countermeasure stage. The communication policy sets the configuration for the communication interval (e.g., 60 s), data replication, data aggregation, multiple path communication, and channel check rate. These communication settings allow the system to perform fine-grained data analysis.

In this work, the DASIF alert policy applies a communication interval of 30 s, data replication, no data aggregation, multiple path communication, and 32 hz as the channel check rate. The multiple path communication is executed using two instances of the IPv6 Routing Protocol for Low Power and Lossy Networks (RPL) protocol. Data are double-communicated via these two instances in order to successfully deliver the notifications even if some node in the path is compromised.

To find two different routes, DASIF forces the RPL instances to select different routes. The main idea for the route selection in the additional instance is: if the main path has the same nodes selected by the secondary path, then DASIF chooses the next best nodes for the secondary path among those devices previously passed in the RPL conditions. The only exception that allows the node parent in both paths is when the node is the sink node (i.e., the path length is 1).

As faults are rare, the normal policy is executed most of the time. This policy sets a communication able to detect the faults and preserve the resources of the devices, i.e., energy.

## 4. Performance Evaluation

DASIF has been evaluated in a simulation environment, considering the related characteristics of an industrial IoT environment. The details of how this evaluation has been conducted are shown in the following sections. Section 4.1 presents the evaluation environment and settings. Section 4.2 describes the metrics used to assess the proposed solution. Section 4.3 presents and discusses the obtained results.

### 4.1. Evaluation Settings

Regarding the dataset, Table 1 shows the settings used to run the tests. The original dataset has 44,643 values for 6 data types: water flow, water temperature, water pressure, steam flow, steam temperature, and steam pressure. The dataset was divided into 70% for training, 20% for tests, and 10% for validation, and it was injected with 446 (1%) faults.

As shown in Matrix (1), the Markov chain states can be maintained, i.e., NN and FF, or a transition can occur, i.e., NF and FN, according to a probability. Matrices (2)–(4) present the Markov chain probabilities used in this evaluation for spike, stuck, and bias faults.

(1) $$Probabilities=\left[\begin{array}{cc} P_{N,N} & P_{N,F}\\ P_{F,N} & P_{F,F} \end{array}\right]$$

(2) $$Stuck=\left[\begin{array}{cc} 0.9966 & 0.0034\\ 0.3 & 0.7 \end{array}\right]$$

(3) $$Spike=\left[\begin{array}{cc} 0.99 & 0.01\\ 1.0 & 0.0 \end{array}\right]$$

(4) $$Bias=\left[\begin{array}{cc} 0.9966 & 0.0034\\ 0.3 & 0.7 \end{array}\right]$$

The machine learning models were implemented in Python using the scikit-learn library [17] running on the Linux operating system. The hardware used for the tests seeks to reflect edge computing hardware. The following hardware was used for these tests: i7-8700 3.2 GHz, 12 GB RAM.

The IoT network has been implemented in Contiki OS and tested in the Cooja simulator [18]. The IoT network has 50 nodes deployed in the area of 100 × 100 m in a grid topology. The RPL multiple instances have been adapted from the solution proposed by Junior et al. [19].

### 4.2. Performance Metrics

The following set of metrics has been defined to measure the performance of the machine learning models:

- **Accuracy**: Indicates the number of correct predictions divided by the total number of predictions.
- **Precision**: Represented by the ratio between the number of correct positive classifications and the number of total positives.
- **Recall**: Represents the number of true positives divided by true positives and false negatives.
- **F1 Score**: Constitutes a harmonic mean between precision and recall. In this metric, 1.0 means excellent performance.

The following metrics assess the IoT network performance:

- **Success of notification delivery**: computed as the division between the number of messages reaching the sink and the total number of sent messages. The duplicated messages count as one notification since they will be eliminated in case of duplicated reception in the sink.
- **Energy consumption**: calculated by the sum of all spent energy of every node during the entire simulation time. This evaluation used the kinetic battery model implemented by Riker et al. [20].
- **Delay**: measures how long it takes to send a message from the IoT node, deliver it to the edge, and detect a fault.

### 4.3. Obtained Results

**Figure 5:**
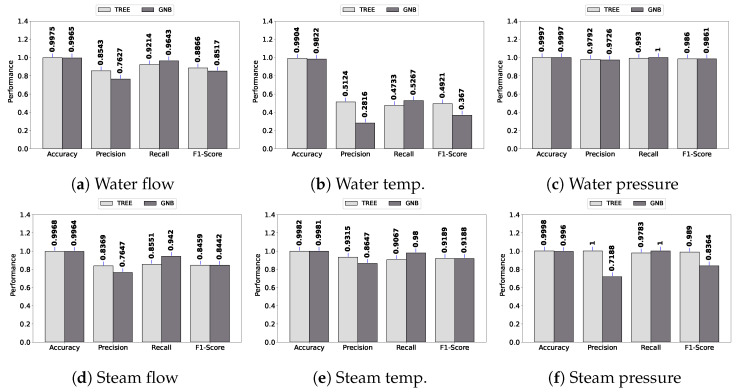
Spike fault.

Figure 5 presents the spike fault detection performance in terms of accuracy, precision, recall, and F1 score.

**Figure 6:**
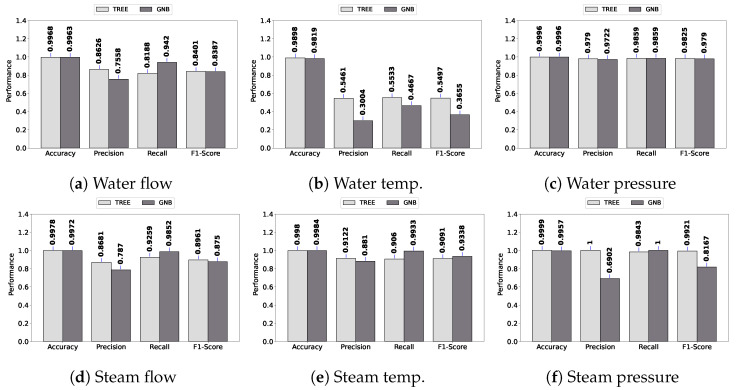
Bias fault.

As can be observed, all models achieve higher than 0.90 when all performance metrics are considered, except for water temperature. A similar performance is observed in Figure 6 for bias fault detection. In general, the performance of fault detection is high. It is important to notice that DASIF considers both classifiers’ outputs in order to detect any fault. This means that if any of the classifiers produce a fault classification, DASIF triggers the state of alert for the system, enforcing the alert communication policy.

Regarding the worst detection performance, i.e., water temperature, the decision tree obtained, on average, 0.54 considering precision, recall, and F1 score for spike faults. For the same datatype and fault type, GNB obtained 0.37. Considering the bias fault, the results for water temperature are 0.49 and 0.38 for the decision tree and GNB, respectively. These results show that the performance in terms of precision, recall, and F1 score is poor for water temperature, but accuracy is high since it is more than 0.98 for both classifiers in spike and bias faults. This occurs because accuracy is a percent value of correct predictions; it is not recommended for an unbalanced dataset. For instance, in a dataset with 1% faults, the accuracy would be 99% if the model predicts zero faults.

**Figure 7:**
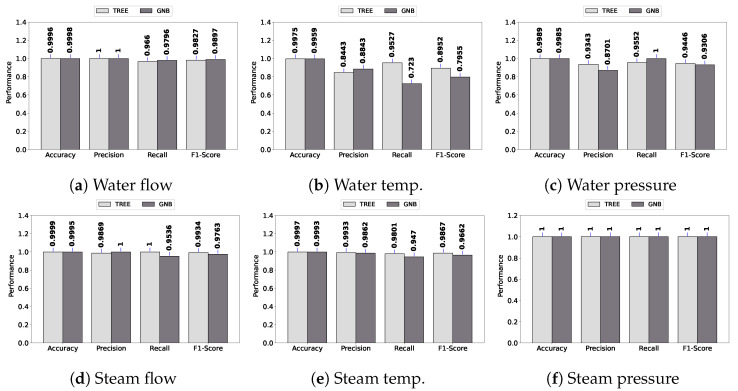
Stuck fault.

Figure 7 shows a higher performance for stuck faults compared to spike and bias. As the stuck fault produces a constant, its faulty pattern can easily be identified by the classifiers.

Table 2 presents the delay results. It is worth mentioning that it refers to the time to communicate and detect a fault. It can be observed that the average delay values are 3.71 and 4.18 for paths 1 and 2, respectively. Path 2 has a higher delay because it tends to select longer paths. The DASIF algorithm for path selection avoids the same nodes selected for path 1. This multiple-path communication strategy enables path diversification, which contributes to higher reliability.

Another aspect in Table 2 is that the time difference between the decision tree and GNB is less than 0.1 milliseconds. Additionally, machine learning classification running on edge computing takes about 2 to 4 milliseconds, while communication is responsible for more than 3.7 to 4.1 s.

Table 3 presents the results of the success of notification delivery and energy. The success rate is 92.76%, considering all communicated application traffic related to the 50 nodes. Regarding energy, the sum of energy consumed by all nodes is 139.17 microA.

## 5. Conclusions and Future Work

Internet of Things and machine learning algorithms applied to industrial environments have caused a revolution in manufacturing systems over the last few years. It is agreed that Industrial Internet of Things can meet some crucial requirements using machine learning and adding intelligence to the continuous monitoring and controlling process. In this context, edge computing is another trend, mainly due to the necessity of achieving low latency on computational tasks in industrial IoT applications.

However, new challenges emerge when the IoT paradigm is added to industrial systems. One of the challenges is that manufacturing systems are prone to faults in IoT devices. These faults represent a risk for the factory since they can result in dangerous events and stop production. Therefore, it is essential to rely on precise and fast systems to detect and react to the occurrence of IoT faults.

This article proposes a framework called Detection and Alert State for Industrial Internet of Things Faults (DASIF). This framework applies machine learning models to detect IIoT faults and also autonomously enforces a state of alert in case of detection of faults. The faults are double classified on the edge computing devices using decision tree and Gaussian naive Bayes. As a reaction, the state of alert enforced by DASIF is the first stage countermeasure for fine-grained data analysis.

In future work, we intend to use federated learning to create a detection system able to run distributively, which can be executed on many industrial sites, maintaining data privacy. Additionally, we will conduct tests in a real environment and design the detection of a more extensive list of faults.

Furthermore, for a functional federated learning solution with heterogeneous data from different machines, semantic schemes and interoperability challenges must be tackled. Some possible approaches are meta-based learning and semantic feature representation.

## Figures and Tables

**Table 1:** Parameters and settings.

Parameter	Value
Original Dataset	44,643 data values; TUPRAS dataset [11]
Data Type	Water flow, water temperature, water pressure
	Steam flow, steam temperature, steam pressure
Type of Fault	Spike, bias, stuck
Injected Faults	446 (1%)
Implementation	Python, scikit-learn library
Edge Computing	Hardware: i7-8700 3.2 GHz, 12 GB RAM
IoT Network	50 nodes

**Table 2:** Delay (seconds) to communicate and detect the fault.

Data Type	Stuck		Spike		Bias	
	TREE	GNB	TREE	GNB	TREE	GNB
**Water flow**						
Path 1	3.7156	3.7157	3.7159	3.7153	3.7162	3.7154
Path 2	4.1839	4.1840	4.1841	4.1835	4.1844	4.1837
**Water temperature**						
Path 1	3.7155	3.7152	3.7157	3.7154	3.7157	3.7154
Path 2	4.1838	4.1835	4.1840	4.1837	4.1839	4.1837
**Water pressure**						
Path 1	3.7157	3.7153	3.7162	3.7155	3.7157	3.7153
Path 2	4.1840	4.1836	4.1844	4.1837	4.1839	4.1836
**Steam flow**						
Path 1	3.7156	3.7153	3.7159	3.7154	3.7160	3.7154
Path 2	4.1839	4.1835	4.1842	4.1837	4.1842	4.1837
**Steam temperature**						
Path 1	3.7156	3.7153	3.7155	3.7155	3.7158	3.7157
Path 2	4.1839	4.1836	4.1838	4.1838	4.1841	4.1839
**Steam pressure**						
Path 1	3.7157	3.7153	3.7156	3.7154	3.7157	3.7156
Path 2	4.1839	4.1836	4.1838	4.1836	4.1840	4.1838

**Table 3:** Success of notification delivery and energy.

Performance	Value
Message Delivery Ratio (%)	92.76
Battery (microA)	139.17

## Data Availability

Not applicable.
